# Clinical Application of the IllumiScan Fluorescence Visualization Device in Detecting Oral Mucosal Lesions

**DOI:** 10.7759/cureus.3111

**Published:** 2018-08-06

**Authors:** Shogo Kikuta, Joe Iwanaga, Keita Todoroki, Katsumi Shinozaki, Ryuichiro Tanoue, Moriyoshi Nakamura, Jingo Kusukawa

**Affiliations:** 1 Dental and Oral Medical Center, Kurume University School of Medicine, Kurume, JPN; 2 Medical Education and Simulation, Seattle Science Foundation, Seattle, USA; 3 Dental and Oral Medical Center, Kurume, JPN

**Keywords:** fluorescence visualization device, illumiscan®, narrow band imaging, oral squamous cell carcinoma, leukoplakia

## Abstract

Objective: Fluorescence visualization devices are screening devices that can be used to examine lesions of the oral mucosa non-invasively. We observed oral squamous cell carcinoma (OSCC) and leukoplakia using the IllumiScan^ ^(Shofu, Kyoto, Japan) fluorescence visualization device and examined its usefulness and characteristics.

Methods: We investigated 31 OSCC and nine leukoplakia in patients who were examined using the IllumiScan and treated in our department from January 2017 to February 2018. Images taken with the IllumiScan were analyzed using image analysis software. We also examined the lesions using narrowband imaging (NBI). Additionally, the IllumiScan and NBI images and the non-stained areas of iodine staining method (IOM) were visually evaluated.

Results: The average luminance of OSCC in the keratinized mucosa was significantly lower than that of OSCC in non-keratinized mucosa. The average luminance of OSCC was significantly lower than that of leukoplakia. Even in keratinized mucosa where IOM is impossible to use, the OSCC lesion exhibited fluorescence visualization loss.

Conclusion: The application of the fluorescence visualization device to the oral mucosa may be useful for distinguishing between cancer and normal areas and can be used to detect OSCC in the keratinized mucosa. The use of the IllumiScan in combination with other conventional screening methods may lead to a better diagnosis.

## Introduction

Oral cancers account for 2.1% of all cancers globally [1]. Oral squamous cell carcinoma (OSCC) makes up approximately 94% of these oral cancers [2]. Oral cancer is one of the few malignant tumors that is directly visible, but in many cases, it is difficult to make a definite diagnosis because of the existence of many oral potentially malignant disorders (OPMD), precancerous lesions, and precancerous conditions in the oral mucosa. Oral cancers present with a wide range of tumor morphologies, and invasive and sometimes painful biopsies may be necessary for a definitive diagnosis. Lugol’s iodine staining method (IOM) is a useful modality that is commonly used to detect OSCC and epithelial dysplasia [3-4]. Previous studies have shown that IOM was useful for detecting oral or esophageal carcinoma and the surrounding epithelial dysplasia [5-6]. However, iodine is irritative to the mucosa, so it cannot be used on keratinized mucosa such as the gingiva and hard palate or in patients with an allergy to iodine [3,7].

Recently, non-invasive and repeatable autofluorescent imaging has been used for screening of the oral mucosa. Autofluorescence arises from the excitation of endogenous fluorescent substances such as flavin adenine dinucleotide (FAD) and collagen crosslinking (CCL) in the oral epithelium and submucosa [8]. Certain fluorescent substances present within the oral epithelium and submucosa fluoresce with a blue excitation (400-460 nm) light source. Oral lesions can be observed through direct fluorescence visualization (FV). FAD and CCL decrease with the progression of the epithelial dysplasia present in SCC and OPMD, resulting in fluorescence visualization loss (FVL), which appears as a dark area on the image [9]. In healthy areas, the blue excitation light source stimulates the emission of green light from endogenous fluorescent substances [10-11].

Optical instruments with fluorescent light, such as the VELscope (LED Dental, White Rock, British Columbia, Canada) and narrow band imaging (NBI; Olympus Corporation, Tokyo, Japan) have been recently used as non-invasive early screening methods, with varying reports about their usefulness [2,12-14]. The IllumiScan (Shofu, Kyoto, Japan) is an alternative fluorescence visualization device, which has a light body and can be used with one hand. Additionally, it can take FV images and save the digital data to the camera. IllumiScan can be used in the complete oral cavity, including the keratinized mucosa, which is not available for IOM. Moreover, unlike VELscope and NBI, this device is compact, convenient to carry, and can be viewed and saved in real time. To our knowledge, there is no study that describes IllumiScan in English to date. In the present study, we investigated the clinical application of the IllumiScan for detecting OSCC and leukoplakia and compared it with other adjunctive screening methods, such as NBI and IOM.

## Materials and methods

Patients

Forty Japanese patients (22 males and 18 females), whose age ranged from 36 to 89 years (mean; 70.4) and who attended the Dental and Oral Medical Center, Kurume University Hospital between January 2017 and February 2018, were examined using a fluorescence visualization device. Of these, 31 patients were pathologically diagnosed with oral squamous cell carcinoma (OSCC) of the tongue (18 cases), gingiva (11 cases), or palate (two cases), and nine patients were clinically diagnosed with leukoplakia. The present study protocol was approved by the ethics committees of our institutions (Approval No. 16147), and the study was performed in accordance with the requirements of the Declaration of Helsinki (64th WMA General Assembly, Fortaleza, Brazil, October 2013).

Fluorescence visualization device and protocol

**Figure 1:**
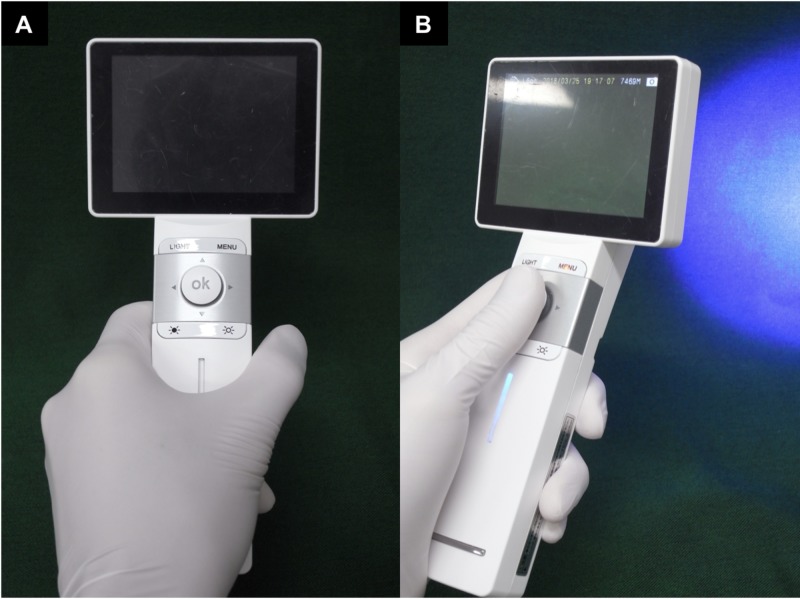
IllumiScan IllumiScan (Shofu, Kyoto, Japan): (A) it has a lightweight body and can be used with one hand; (B) it irradiates a blue excitation light source (425 nm). A fluorescence visualization image can save the digital data to the camera attached to the device and be observed in real time.

In this study, the IllumiScan fluorescence visualization device was used to examine the oral mucosa (Figure 1). This instrument takes fluorescence visualization (FV) images and saves the digital data to the camera. The FV images of a target area can be observed in real time because the instrument has its own monitor. It has a lightweight body and can be used with one hand. FV images were taken in a darkened room, with a distance between the target area and the device of about 15 cm. FV images from the IllumiScan were analyzed using Image J software (version 1.5; National Institutes of Health, Bethesda, MD, USA). A region of interest (ROI) for a target and a control area were established in the FV images. The observer first set the boundary of the clinical tumor on a visual examination of the intraoral images, and then the area of viewed FV loss was determined using the IllumiScan. The region of interest (ROI) for a target area was defined as the area including the two areas [15-16]. The ROI for the control area was set near the ROI for a target area at the same site without an induration of the tumor. FV images were divided into RGB components (R: Red, G: Green, B: Blue) and the green image was extracted. The average luminance of the ROI for the green image was defined as the G value (cd/m2). The luminance of the target area divided by the luminance of the control area was defined as the luminance ratio (LR). The coefficient of variation (CV; the standard deviation divided by the mean) was calculated for the area (pixels), G value (cd/m2), and LR.

Narrow band imaging (NBI) magnifying endoscopy

The Evis Lucera Spectrum (Olympus Medical System Corp., Tokyo, Japan), a magnifying endoscopy system with narrow band imaging (NBI), was used. This system comprises a video processor with a special optical filter (CV-260SL), a light source (CLV-260SL), and a videoscope using GIF-H260Z. The subjects consisted of 25 patients who were diagnosed with OSCC (16 in the tongue, 9 in the gingiva) following clinical evaluation. The target images visualized by NBI were classified into four types according to Takano’s classification (types 1–4) for oral mucosal lesions [17]. Type 1 (normal) is both waved arms together, as a waved line.; and type 2 is a similar shape to type 1, but their caliber is remarkably increased compared with others far from the lesion. Type 3 is a simple increase in length or as tangled lines due to the severe increase in length.; while type 4 is characterized by large vessels with no loops at the terminal. Each patient was classified and analyzed for correlation with fluorescence luminance.

Iodine staining method (IOM)

OSCCs and leukoplakias of the tongue were dried with gauze, and 10% Lugol’s iodine solution was applied and immediately washed off. The area unstained by Lugol’s solution was observed after the application and recorded. The area stained by Lugol’s solution was defined as the normal epithelial area, and the unstained area was defined as the epithelial dysplasia area. This image was later visually compared with other images taken using the IllumiScan and NBI.

Statistical analysis

All quantitative results were expressed as the mean ± SD. One-way analysis of variance with Scheffé’s post hoc test and Fisher’s exact test was used to compare the data. Statistical significance was set at p < 0.05, unless otherwise indicated. Receiver operating characteristic (ROC) curves were created using JMP statistical software (Version 13.2, SAS Institute Inc, NC, USA). The area under the curve (AUC), sensitivity, and specificity were calculated by a ROC analysis. The cutoff value was defined according to the Youden index [18].

## Results

Fluorescence visualization analysis

**Figure 2:**
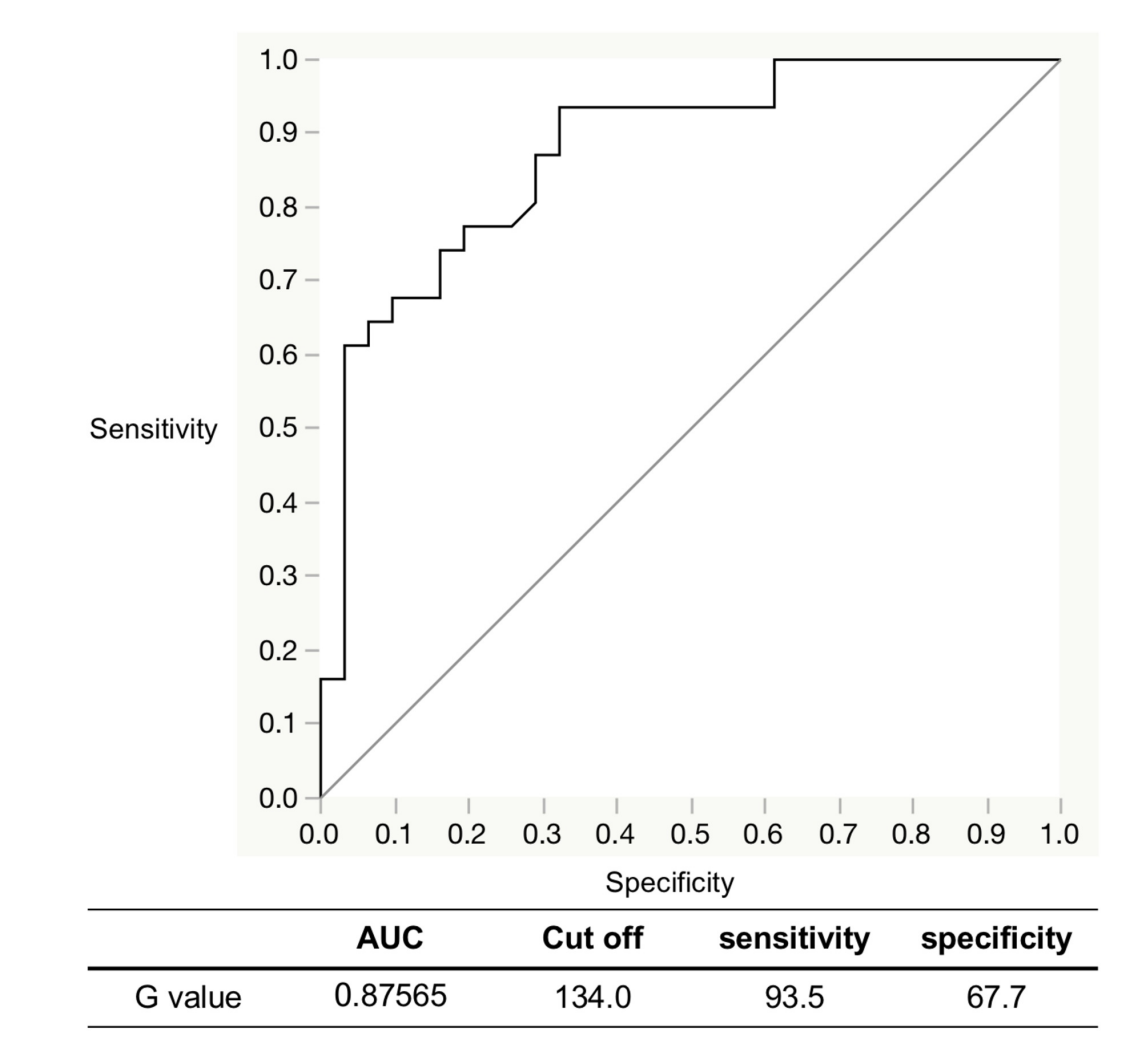
Evaluation of detection accuracy by ROC analysis with IllumiScan of OSCC and the control ROC: receiver operating characteristic; OSCC: oral squamous cell carcinoma; AUC: area under the curve

The area of the ROI for OSCC ranged from 11606.0 to 243651.0 pixels (mean: 69473.1 ± 55255.8 pixels, CV = 0.8). The area of the ROI for the control ranged from 1804.0 to 2053.0 pixels (mean: 1979.2 ± 42.0 pixels, CV = 0.02). There was a statistically significant difference between both (n = 31, p < 0.05, p = 0.0001 one-way analysis of variance with Scheffé’s post hoc test). The G value of the ROI for OSCC ranged from 46.0 to 152.7 cd/m2 (mean: 93.4 ± 28.3 cd/m2, CV = 0.3). The G value of the ROI for the control ranged from 105.8 to 222.1 cd/m2 (mean: 145.9 ± 35.0 cd/m2, CV = 0.24). There was a statistically significant difference between both (n = 31, p < 0.05, p = 2.7 E-8). On the created ROC curve for G values, the maximal point of addition of sensitivity and specificity was 134.0 cd/m2, which was defined as the cutoff value for the diagnosis of OSCC for the ROI area (Figure 2).

The area of the ROI for OSCC in the non-keratinized and keratinized mucosa ranged from 17295.0 to 243651.0 pixels (n = 18, mean: 88695.8 ± 60458.4 pixels, CV = 0.68) and 11606.0 to 109279.0 pixels (n = 13, mean: 42856.9 ± 31616.3 pixels, CV = 0.74), respectively. There was a statistically significant difference between both (p < 0.05, p = 0.013). The G value of the ROI for OSCC in the non-keratinized and keratinized mucosa ranged from 46.5 to 152.7 cd/m2 (n = 18, mean: 104.6 ± 27.9 cd/m2, CV = 0.27) and 46.0 to 121.0 cd/m2 (n = 13, mean: 78.0 ± 20.3 cd/m2, CV = 0.26), respectively. There was a statistically significant difference between both (p < 0.05, p = 0.008). The LR values of the ROI for OSCC in the non-keratinized and keratinized mucosa were 0.67 ± 0.13 (CV = 0.27) and 0.62 ± 0.2 (CV = 0.29), respectively, and the difference between them was not significant (p > 0.05, p = 0.43). Next, we analyzed FV in the growth pattern and cancer staging of OSCC. There were no statistically significant differences among the three types of growth patterns and the early/late stage (Tables 1-2). However, the G value of the late stage tended to be lower than that of the early stage in the single regression analysis.

**Table 1:** Fluorescence visualization analysis of the growth pattern CV: coefficient of variation

	Superficial Type (n = 12)	Exophytic Type (n = 10)	Endophytic Type (n = 9)
Area（pixels）	59845.0 ± 43218.2 (11687.0 – 143863.0)	76420.6 ± 66808.8 (11606.0 – 243651.0)	74591.0 ± 53510.8 (16012.0 – 171569.0)
CV	0.72	0.87	0.72
G value (cd/m^2^)	94.2 ± 31.1 (51.6 – 152.7)	102.8 ± 30.0 (46.0 – 134.0)	82.1 ± 15.4 (63.2 – 112.7)
CV	0.33	0.29	0.19
Luminance ratio	0.67 ± 0.19	0.66 ± 0.10	0.61 ± 0.10
CV	0.28	0.23	0.13

**Table 2:** Fluorescence visualization analysis of the cancer staging CV: coefficient of variation

	Early stage (n = 23)	Late stage (n = 8)	P value
Area（pixels）	70129.8 ± 55305.0 (11687.0 – 243651.0)	67585.0 ± 55070.5 (16012.0 – 171569.0)	
CV	0.79	0.81	
G value (cd/m^2^)	98.7 ± 27.6 (46.5 – 152.7)	78.2 ± 24.3 (46.0 – 134.0)	0.08
CV	0.28	0.31	
Luminance ratio	0.67 ± 0.16	0.59 ± 0.11	0.2
CV	0.24	0.20	

**Figure 3:**
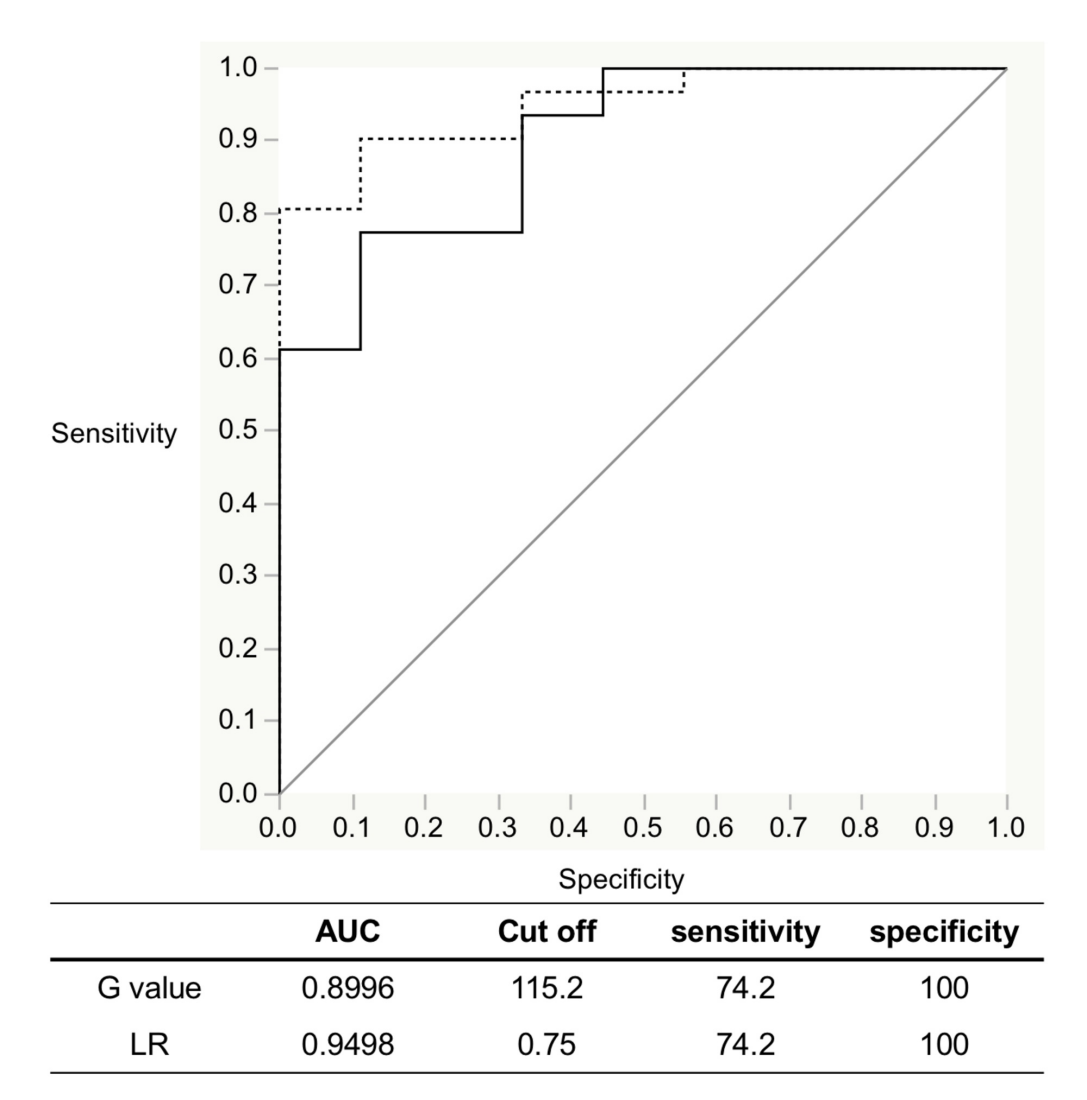
Evaluation of detection accuracy by ROC analysis with IllumiScan of OSCC and leukoplakia ROC: receiver operating characteristic; OSCC: oral squamous cell carcinoma; AUC: area under the curve; Dotted line: Luminance ratio (LR); Black line: G value

The area of the ROI for leukoplakia ranged from 7716.0 to 85818.0 pixels (n = 9, mean: 29054.0 ± 22191.2 pixels, CV = 0.76). There was a statistically significant difference between OSCC and leukoplakia (p < 0.05, p = 0.003). The G value of the ROI for leukoplakia ranged from 94.6 to 191.2 cd/m2 (n = 9, mean: 146.6 ± 31.5 cd/m2, CV = 0.21). There was a statistically significant difference between OSCC and leukoplakia (p < 0.05, p = 0.00003). The LR of the ROI for leukoplakia was 1.9 ± 0.14 (CV = 0.14). There was a statistically significant difference between OSCC and leukoplakia (p < 0.05, p = 0.000001). On the created ROC curve for G value and LR, the maximal point of addition of sensitivity and specificity was 115.2 cd/m2 and 0.75, respectively, which was defined as the cutoff value for the criterion to distinguish between OSCC and leukoplakia. The AUC was 0.900 for the G value and 0.950 for the LR, demonstrating a high degree of accuracy (Figure 3).

**Figure 4:**
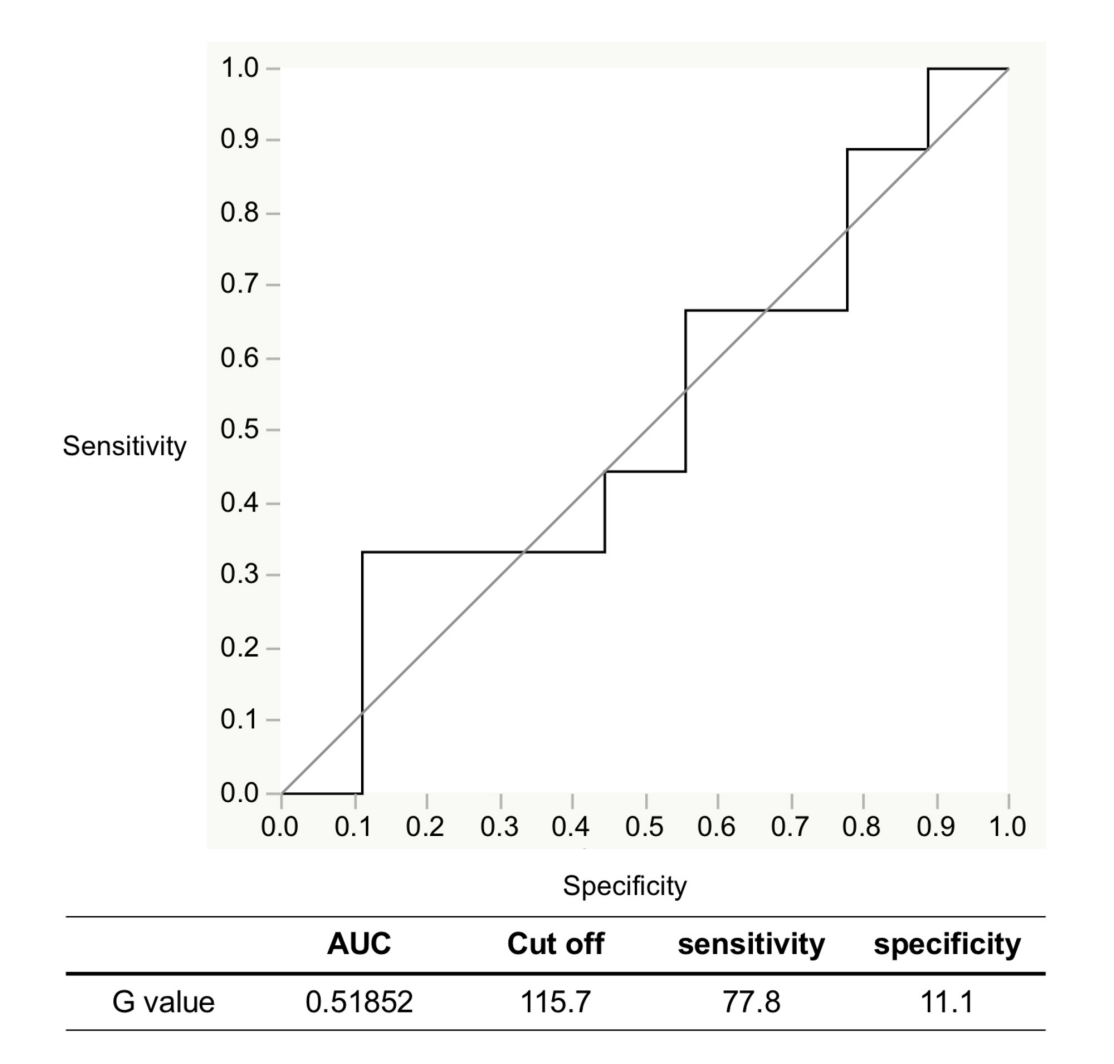
Evaluation of detection accuracy by ROC analysis with IllumiScan of leukoplakia and the control ROC: receiver operating characteristic; AUC: area under the curve

The comparison between leukoplakia and the control for the ROI area resulted in an AUC of 0.518, indicating a low degree of accuracy (Figure 4).

Comparison between NBI and FV analyses

Twenty-six of the 31 OSCC cases were examined using NBI. According to Takano’s classification, there were 16 type 3 cases (61.5%), 10 type 4 cases (38.5%), and the G value of the type 4 cases tended to be lower than that of the type 3 cases in the single regression analysis.

Comparison and detection of the ROI area in each visual image

**Figure 5:**
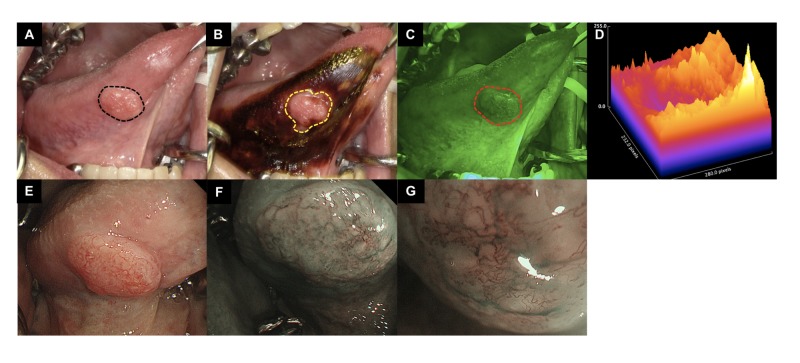
Comparison of tongue SCC observed with each examination method (macroscopy, IOM, IllumiScan, NBI) (A) clinically apparent tumor outlined in black dotted line; (B) iodine unstained area by IOM outlined in yellow dotted line; (C) FV loss area outlined in red dotted line; (D) Surface plot of tumor area visualized by Image J; (E) tumor image by ordinary light; (F) observation by NBI (low power field); (G) observation by NBI (high power field), the irregularly multilayered and branched reticular vessels was showed, which is Type 4 of Takano's classification. SCC: squamous cell carcinoma; IOM: iodine staining method; NBI: narrowband imaging; FV: fluorescence visualization

The images of lesions observed with each examination method (macroscopy, IOM, IllumiScan, NBI) were compared. Case 1 was an exophytic-type OSCC of the tongue (T1N0M0). The macroscopic lesion was consistent with Lugol’s unstained area and the FVL area. Additionally, the NBI images revealed strong FVL in an area showing irregularly branched, reticular vessels (Fig. 5). 

**Figure 6:**
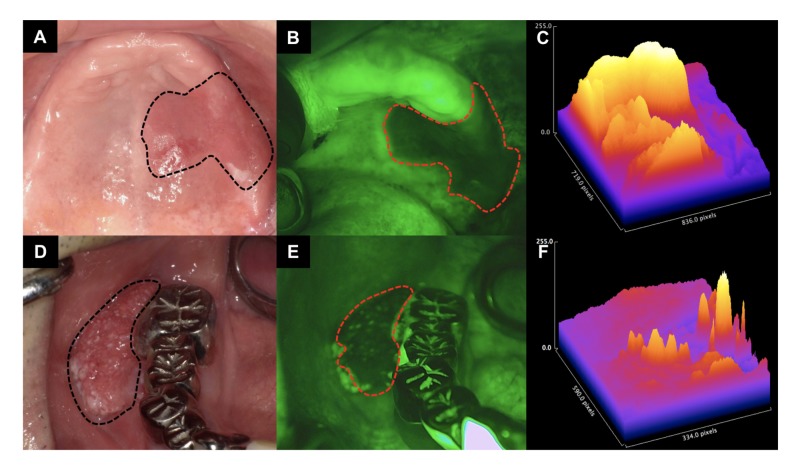
Observation of palatal and gingival SCC observed with each examination method (macroscopy, IllumiScan) (A) clinically apparent tumor in palatal mucosa outlined in black dotted line; (B) FV loss area outlined in red dotted line; (C) Surface plot of tumor area visualized by Image J; (D) clinically apparent tumor in gingival mucosa outlined in black dotted line; (E) FV loss area outlined in red dotted line; (F) surface plot of tumor area visualized by Image J. SCC: squamous cell carcinoma; FV: fluorescence visualization

Case 2 was a superficial-type OSCC of the tongue (T1N0M0). The Lugol’s unstained area was not consistent with the FVL area. The area that did not show FV loss corresponded to the white area of the lesion. In the gingival and palatal areas, in which IOM could not be used, the lesion was relatively clearly delineated by the IllumiScan observation (Figure 6).

## Discussion

Several studies have reported using oral mucosal fluorescence visualization devices, raising much speculation about their usefulness and role in the detection of oral mucosal lesions [2,7-8,12,15-16]. The IllumiScan is the first fluorescence visualization device for the oral mucosa to be made in Japan. The IllumiScan has a light body, can be operated with one hand, and can be used to take FV images while saving the digital data inside the camera. We studied the clinical application of the IllumiScan in the detection of oral mucosal lesions and compared it with other screening methods. Another fluorescence visualization device, the VELscope, uses a blue excitation light source (400-460 nm), making it difficult to observe the dorsum of the tongue, which emits red light because of abundant porphyrin. Koch et al. previously reported that a lesion in the dorsum of the tongue would not be detected by the VELscope [19]. However, the IllumiScan uses a blue excitation light source (425 nm) and allows the observation of the whole oral cavity by cutting out the red light emitted by porphyrin [16]. Epithelial dysplasia causes a decrease in FAD and CCL, which are the main sources of the excitation of endogenous fluorescent substances in the oral epithelium and submucosa [9]. The decrease in FAD and CCL causes continuous FVL. Additionally, hemoglobin within blood vessels strongly absorbs blue excitation light [11]. Epithelial dysplasia and cancer lesions promote metabolic turnover and increase tumor neovascularization and vessel density. Moreover, inflammatory lesions expand peripheral blood vessels [16]. These multiple factors result in FVL.

Yamamoto et al. investigated the detection accuracy of the objective autofluorescence visualization method with EZR statistical software (Saitama Medical Center Jichii Medical University, Saitama, Japan) [20] and found that the diagnosis of epithelial dysplasia of the tongue using the VELscope resulted in 90.6% sensitivity and 80% specificity [12]. Morikawa et al. reported that objective evaluations of the G value and LR with Image J software (version 1.5; National Institutes of Health, Bethesda, MD, USA) using IllumiScan to diagnose SCC and leukoplakia of the tongue resulted in 90% and 100% sensitivity and 66.7% and 88.9% specificity, respectively [21]. They also reported that objective evaluations with Image J software for diagnosis of OSCC and oral lichen planus resulted in 61.0% and 59.3% sensitivity and 78.0% and 100% specificity, respectively [16]. In the present study, objective evaluations of the G value using the IllumiScan for the diagnosis of OSCC and the control resulted in 93.5% sensitivity and 67.7% specificity. These results suggest that using the IllumiScan may be useful for distinguishing between normal areas and OSCC. The G value of OSCC in the keratinized mucosa not available for IOM was significantly lower than that of OSCC in non-keratinized mucosa. The objective evaluations of G value and LR using the IllumiScan for the diagnosis of OSCC and leukoplakia showed that sensitivity and specificity were concordantly 74.2% and 100%, respectively. However, the objective evaluations of G value using the IllumiScan for normal areas and leukoplakia showed that sensitivity and specificity were 77.8% and 11.1%, respectively. The area of leukoplakia strongly reflects the blue excitation light because of its thick keratinized layer, so it may be difficult to distinguish both areas using the IllumiScan. Recently, Kwan et al. described the intratumoral heterogeneity of oral cancer, which reflects not only its biologic constituents, but also its gene expression, metabolic, and behavioral characteristics [22]. The results of CV in this study are indicative of intratumoral heterogeneity.

## Conclusions

In conclusion, the application of a fluorescence visualization device to the oral mucosa may be useful for distinguishing between cancer and normal areas. The device can be used to detect OSCC in the keratinized mucosa, but an accurate identification of the lesion area may be difficult. Combining the simple and convenient IllumiScan device with other conventional screening methods may lead to a better diagnosis.

## References

[1] Ferlay J, Soerjomataram I, Dikshit R (2015). Cancer incidence and mortality worldwide: sources, methods and major patterns in GLOBOCAN 2012. Int J Cancer.

[2] McNamara KK, Martin BD, Evans EW, Kalmar JR (2012). The role of direct visual fluorescent examination (VELscope) in routine screening for potentially malignant oral mucosal lesions. Oral Surg Oral Med Oral Pathol Oral Radiol Endod.

[3] Kuribayashi Y, Tsushima F, Sato M, Morita K, Omura K (2012). Recurrence patterns of oral leukoplakia after curative surgical resection: important factors that predict the risk of recurrence and malignancy. J Oral Pathol Med.

[4] McMahon J, Devine JC, McCaul JA, McLellan DR, Farrow A (2010). Use of Lugol’s iodine in the resection of oral and oropharyngeal squamous cell carcinoma. Br J Oral Maxillofac Surg.

[5] Kurita H, Kurashina K (1996). Vital staining with iodine solution in delineating the border of oral dysplastic lesions. Oral Surg Oral Med Oral Pathol Oral Radiol Endod.

[6] Sugimachi K, Kitamura K, Baba K, Ikebe M, Kuwano H (1992). Endoscopic diagnosis of early carcinoma of the esophagus using Lugol’s solution. Gastrointest Endosc.

[7] López-Jornet P, De la Mano-Espinosa T (2011). The efficacy of direct tissue fluorescence visualization in screening for oral premalignant lesions in general practice: an update. Int J Dent Hyg.

[8] Awan KH, Morgan PR, Warnakulasuriya S (2011). Evaluation of an autofluorescence based imaging system (VELscopeTM) in the detection of oral potentially malignant disorders and benign keratoses. Oral Oncol.

[9] Drezek R, Brookner C, Pavlova I (2001). Autofluorescence microscopy of fresh cervical-tissue sections reveals alterations in tissue biochemistry with dysplasia. Photochem Photobiol.

[10] De Veld DC, Witjes MJ, Sterenborg HJ, Roodenburg JL (2005). The status of in vivo autofluorescence spectroscopy and imaging for oral oncology. Oral Oncol.

[11] Richards-Kortum R, Sevick-Muraca E (1996). Quantitative optical spectroscopy for tissue diagnosis. Annu Rev Phys Chem.

[12] Yamamoto N, Kawaguchi K, Fujihara H (2017). Detection accuracy for epithelial dysplasia using an objective autofluorescence visualization method based on the luminance ratio. Int J Oral Sci.

[13] Iwamoto O, Kusukawa J (2013). Observation of stage Ⅰ and stage Ⅱ oral squamous cell carcinoma using an endoscope with a built-in special light system (NBI, AFI, IRI) [Article in Japanese]. J Jpn Soc Oral Oncol.

[14] Shibahara T, Yamamoto N, Yakushiji T, Nomura T, Sekine R, Muramatsu K, Ohata H (2014). Narrow-band imaging system with magnifying endoscopy for early oral cancer. Bull Tokyo Dent Coll.

[15] Poh CF, Zhang L, Anderson DW (2006). Fluorescence visualization detection of field alterations in tumor margins of oral cancer patients. Clin Cancer Res.

[16] Morikawa T, Bessho H, Kozakai A, Kosugi A, Shibahara T (2017). Analysis of oral squamous cell carcinoma and oral lichen planus using the “IllumiScan” optical instrument [Article in Japanese]. The Shikwa Gakuho.

[17] Takano JH, Yakushiji T, Kamiyama I, Nomura T, Katakura A, Takano N, Shibahara T (2010). Detecting early oral cancer: narrowband imaging system observation of the oral mucosa microvasculature. Int J Oral Maxillofac Surg.

[18] Fluss R, Faraggi D, Reiser B (2005). Estimation of the Youden Index and its associated cutoff point. Biom J.

[19] Koch FP, Kaemmerer PW, Biesterfeld S, Kunkel M, Wagner W (2011). Effectiveness of autofluorescence to identify suspicious oral lesions-a prospective, blinded clinical trial. Clin Oral Investig.

[20] Kanda Y (2013). Investigation of the freely available easy-to-use software “EZR” for medical statistics. Bone Marrow Transplant.

[21] Morikawa T, Kosugi A, Bessho H, Nomura T, Katakura A, Shibahara T (2017). Analysis of tongue squamous cell carcinoma and leukoplakia by optical instrument. J Jpn Stomatol Soc.

[22] Kwon SH, Yoon JK, An YS (2014). Prognostic significance of the intratumoral heterogeneity of 18F-FDG uptake in oral cavity cancer. J Surg Oncol.

